# Automated manufacturing and characterization of clinical grade autologous CD20 CAR T cells for the treatment of patients with stage III/IV melanoma

**DOI:** 10.3389/fimmu.2024.1328368

**Published:** 2024-09-25

**Authors:** Krasimira Aleksandrova, Jana Leise, Christoph Priesner, Murat Aktas, Michael Apel, Mario Assenmacher, Iris Bürger, Anne Richter, Pia Altefrohne, Christine Schubert, Astrid Holzinger, Markus Barden, Valerie Bezler, Michael von Bergwelt-Baildon, Peter Borchmann, Lilia Goudeva, Wolfgang Glienke, Lubomir Arseniev, Ruth Esser, Hinrich Abken, Ulrike Koehl

**Affiliations:** ^1^ Institute of Cellular Therapeutics (ICT), Hannover Medical School (MHH), Hanover, Germany; ^2^ Miltenyi Biotec B.V. & Co. KG, Bergisch Gladbach, Germany; ^3^ Miltenyi Biomedicine GmbH, Bergisch Gladbach, Germany; ^4^ Division of Genetic Immunotherapy, Leibniz Institute for Immunotherapy (LIT) and University of Regensburg, Regensburg, Germany; ^5^ Medical Department III, Hospital of the Ludwig Maximillian University of Munich (LMU), Munich, Germany; ^6^ Internal Medicine, University Hospital Cologne, Cologne, Germany; ^7^ Institute of Transfusion Medicine and Transplant Engineering (ITMTE), Hannover Medical School, Hannover, Germany; ^8^ Fraunhofer Institute for Cell Therapy and Immunology (IZI), Leipzig, Germany; ^9^ Institute of Clinical Immunology, University of Leipzig, Leipzig, Germany

**Keywords:** clinical CD20 CAR T cell trial, automated manufacturing of engineered T cell products, ex vivo expansion, cell composition of CAR T cell products, CD20 CAR investigational medicinal product

## Abstract

**Introduction:**

Point-of-care (POC) manufacturing of chimeric antigen receptor (CAR) modified T cell has expanded rapidly over the last decade. In addition to the use of CD19 CAR T cells for hematological diseases, there is a growing interest in targeting a variety of tumor-associated epitopes.

**Methods:**

Here, we report the manufacturing and characterization of autologous anti-CD20 CAR T cells from melanoma patients within phase I clinical trial (NCT03893019). Using a second-generation lentiviral vector for the production of the CD20 CAR T cells on the CliniMACS Prodigy®.

**Results:**

We demonstrated consistency in cell composition and functionality of the products manufactured at two different production sites. The T cell purity was >98.5%, a CD4/CD8 ratio between 2.5 and 5.5 and transduction rate between 34% and 61% on day 12 (harvest). Median expansion rate was 53-fold (range, 42–65-fold) with 1.7-3.8×10^9^ CAR T cells at harvest, a sufficient number for the planned dose escalation steps (1×10^5^/kg, 1×10^6^/kg, 1×10^7^/kg BW). Complementary research of some of the products pointed out that the CAR+ cells expressed mainly central memory T-cell phenotype. All tested CAR T cell products were capable to translate into T cell activation upon engagement of CAR target cells, indicated by the increase in pro-inflammatory cytokine release and by the increase in CAR T cell amplification. Notably, there were some interindividual, cell-intrinsic differences at the level of cytokine release and amplification. CAR-mediated T cell activation depended on the level of CAR cognate antigen.

**Discussion:**

In conclusion, the CliniMACS Prodigy® platform is well suited for decentralized POC manufacturing of anti-CD20 CAR T cells and may be likewise applicable for the rapid and automated manufacturing of CAR T cells directed against other targets.

**Clinical trial registration:**

https://clinicaltrials.gov/study/NCT03893019?cond=Melanoma&term=NCT03893019&rank=1, identifier NCT03893019.

## Introduction

The incidence of melanoma is increasing worldwide, and despite early detection and intervention, the number of patients dying from metastatic disease continues to rise. However, during the last decade, the use of immune checkpoint blocking antibodies and targeted drugs like BRAF inhibitors in patients with stage III/IV melanoma improved progression-free survival and overall survival compared to previous chemotherapy (1–8). In addition, there is a rationale for targeting CD20, as melanoma lesions harbor CD20+ melanoma cells with stem-like properties. Schlaak et al. demonstrated that lesional injections of anti-CD20 monoclonal antibody Rituximab induced metastatic melanoma regression in a patient (9). To achieve a long-term effect, chimeric antigen receptor (CAR) T cells redirected against CD20 may be a good option to obtain long-term cancer control like targeting CD19 in hematological diseases (10–15).

Previously, it was demonstrated (16) in a transplant mouse model that selective elimination of melanoma cell population by CAR T cells is particularly effective in eradicating established melanoma lesions irrespective of the tumor size. CAR T cells engineered to recognize HMW-MAA (MCSP), which is broadly expressed by the majority of cancer cells in melanoma, reduced tumors in xenograft models. Of note, T cells engineered with a CD20-specific CAR likewise eradicated melanoma, although <2% of melanoma cells expressed CD20, along with HMW-MAA. Since targeting of any random 10% tumor cell subset was not effective, there is a strong rationale that maintenance and/or progression of melanoma depends on the minor CD20+ subset of melanoma cells. The concept is in line with the identification of CD20+ melanoma stem cells (17) and the “stem cell paradigm” for tumor progression (18–20). On that basis, a phase I clinical trial was designed to evaluate the efficacy of anti-CD20 CAR T cells in the treatment of metastatic melanoma (NCT03893019).

The canonical CAR currently used in clinical applications is a “second generation” CAR composed of an extracellular binding domain for cognate antigen, mostly a single chain fragment of variable region (scFv) antibody, followed by a spacer domain, frequently derived from the IgG1 constant domain, and the transmembrane and intracellular costimulatory domain linked to the intracellular CD3ζ signaling moiety. The costimulatory domain is derived from CD28 or 4-1BB, both required to enhance and prolong CD3ζ mediated T-cell activation, however, displaying different activation properties (21). CD28 costimulatory CARs provide rapid T-cell activation with accelerated entry into a dysfunctional state (“exhaustion”), while 4-1BB co-stimulation triggers a moderate but protracted T-cell activation.

Second-generation CAR directed against CD20 with 4-1BB costimulatory domain was chosen as investigational medicinal product (IMP) for the clinical trial. The previously reported protocols for establishing and validation of the manufacturing using healthy donor apheresis as a starting material (22, 23) have been applied for the clinical-scale automated production of the IMP. Here, we report the results of the manufacturing and characterization of the autologous anti-CD20 CAR T cells and demonstrate consistency in composition and functionality of the products manufactured at two different manufacturing sites.

## Materials and methods

### Patients and manufacturing sites

A total of 15 female and male patients over 18 years with unresectable stage III or IV melanoma and progressive disease were to be enrolled in a phase I multi-centric, single-arm, prospective, open, dose-escalation trial (NCT03893019) for the treatment with the IMP “MB-CART20.1”, i.e., anti-CD20 CAR T-cell product. Finally, nine patients (seven male and two female) were assigned to two different dosage levels of the IMP. The current diagnosis at screening of all patients was AJCC V8 disease stage IV. All patients had received a previous immunotherapy, two patients had a previous chemotherapy, four patients had received targeted therapy drugs, and four patients had a previous radiation therapy. All patients had findings in medical history, the most frequently reported diseases were endocrine and gastrointestinal disorders. Blood samples for routine laboratory parameters and for persistence and cytokine analysis were collected regularly during the course of the trial.

Patients were pre-treated with 60 mg/kg body weight cyclophosphamide (day −7 and day −6) and 25 mg/m^2^ body surface area fludarabine (day −5 to day −1) before intravenous infusion of MB-CART20.1 on day 0, which corresponds day 13 of the manufacturing of the IMP.

The IMPs were produced at two manufacturing sites. Five of the nine products were manufactured at the Institute of Cellular Therapeutics (ICT, department Cellular Therapy Centre), Hannover Medical School, Hanover, Germany and the other four at Miltenyi Biotec (MB), Bergisch-Gladbach, Germany.

### CAR construct

The lentiviral expression cassettes encoding for the CD20-specific CAR used in this study were obtained by engineering the Leu16 scFv (24) with specificity to CD20 onto a prototype second-generation CAR backbone scFv-CD8a-4-1BB-CD3ζ previously described by Lock et al. (25). The construct design was based on a retroviral anti-CD20 CAR vector originally described by Schmidt et al. (16). The transducing vector encoding the anti-CD20 CAR was engineered and produced by Lentigen Technology, Inc. (Gaithersburg, MD, USA) and provided by Miltenyi Biotec under the product name “Lentiviral Vector (anti-) CD20 CAR 10 mL per bag”.

### Selection, stimulation, transduction, and expansion via CliniMACS Prodigy®

CliniMACS Prodigy® (Miltenyi Biotec) was selected for the production of anti-CAR-T cells as a scalable, robust platform designed to enable automated and decentralized manufacturing of various cell types in a closed system (26, 27).

The manufacturing followed the validated manufacturing protocol previously reported by (23). One deviation was that the starting materials, i.e., peripheral blood cells, were collected by leukapheresis from study patients instead of healthy donors on day −1 of the production process. All further steps of the manufacturing were carried out using the automated CliniMACS Prodigy® platform and tubing set 520 (both Miltenyi Biotec) under GMP conditions in a class A/B clean room at ICT and class C clean room at MB, respectively. CD4+ and CD8+ T cells were magnetically enriched from the leukapheresis using the GMP-grade CliniMACS^®^ CD4+ and CD8+ Microbeads (Miltenyi Biotec). Enriched T cells (1×10^8^) were activated and expanded by incubation with stimulating CD3/CD28 antibodies (“MACS^®^ GMP T cell TransAct”, Miltenyi Biotec) in TexMACS™ GMP Medium supplemented with MACS^®^ GMP Recombinant Human IL-7 and MACS^®^ GMP Recombinant Human IL-15 (all Miltenyi Biotec). The transducing lentiviral vector encoding the anti-CD20 CAR was added to the cell culture within 16–24 h after T-cell activation. The culture medium was supplemented with 3% (v/v) AB serum [“Pool-Humanserum/AB PHS/Tu”, Centre for Clinical Transfusion Medicine Tubingen (ZKT Tübingen gGmbH), Tubingen, Germany] for the first 5 days; the cells were further incubated in serum-free medium for the remaining 7 days. The IMP was formulated accordingly to the respective dosage in electrolyte storage solution [“Composol^®^ PS”, Fresenius Kabi Deutschland GmbH, Bad Homburg, Germany supplemented with 2.5% (w/v) Human Albumin (w/v), Octapharma GmbH, Springe, Germany or “CliniMACS^®^ Formulation Solution”, Miltenyi Biotec].

### In-process and Quality control

The in-process controls (IPCs) at day 0 (start of manufacturing process) provided the basis for the manufacturing settings. The next IPC on day 5 served as status check of production success immediately before the patient’s lymphodepletion at day 6 (which corresponds day −7 before intravenous infusion of the IMP). Samples for microbiological control (mycoplasmas, bacteria, and fungi) were drawn on day 10 to be able to obtain preliminary results at the time of the IMP release on day 12. The final QC was performed on the harvested product on day 12 followed by dosage confirmation after the final formulation of the IMP. The IPC/QC test points, methods, and acceptance criteria are listed in **Table 1**. All applied methods were validated matrix specifically in accordance with the relevant Ph. Eur. general methods of analysis or/and under consideration of the scientific guideline ICH Q2(R2) Validation of analytical procedures.

**Table 1:** Time points and specifications for decisive in-process controls (IPC) and quality controls (QC).

Parameter	IPC Time Point Acceptance Criteria	QC Time Point Acceptance Criteria	Method
CD3+ cell viability	Day 5 determined/declared	Day 12 (at harvest) ≥80% viable cells among CD45+CD3+ cells	Flow cytometry Ph. Eur. 2.7.24
CD3+ cell percentage	Day 5 determined/declared	Day 12 (at harvest) ≥80% CD3+ cells among viable CD45+ cells	Flow cytometry Ph. Eur. 2.7.24
Transduction Frequency	Day 5 ≥5% CAR+ cells among viable CD3+ cells	Day 12 (at harvest) ≥10% CAR+ cells among viable CD3+ cells	Flow cytometry Ph. Eur. 2.7.24
Identity CD3+CAR+ cells/ Potency	Day 5 CD20 CAR Detection Reagent bound to viable CD3+ cells	Day 12 (at harvest) CD20 CAR Detection Reagent bound to viable CD3+ cells	Flow cytometry Ph. Eur. 2.7.24
Dose (CD3+CAR+ cells/mL)	Day 5 determined/declared	Day 12 IMP according to cohort dosage deviation of 20% is acceptable	Flow cytometry Ph. Eur. 2.7.24
Appearance	/	Day 12 IMP slightly turbid infusion dispersion, primary package integrity	Visual inspection
Endotoxin	Day 5 determined/declared	Day 12 IMP <5 EU/mL	LAL Method D Ph. Eur. 2.6.14
Microbiological Examination	Day 10 negative-to-date	day 12 IMP negative (result obtained post-release)	Ph. Eur. 2.6.27
Mycoplasma	Day 10 negative-to-date	Day 12 IMP negative (result obtained post-release)	Ph. Eur. 2.6.7
Vector Copy Number	/	Day 12 (at harvest) <5 copies per transduced cell (result obtained post-release)	Real-time qPCR

#### Flow cytometric analyses

Flow cytometric analyses of the cell composition, identity, purity, and transduction frequency and dosage determination were performed as previously described in detail (23) on day 0, day 5, and on harvest at day 12 using the flow cytometer MACSQuant Analyzer 10 (Milteny Biotec). Briefly, absolute cell counts were determined volumetrically, calculated automatically, and read out as counts/μL. Viability of the cells was determined by use of 7-amino actinomycin D (7-AAD) to stain non-viable cells. Lyse/no-wash method was applied for the cell staining with monoclonal antibodies against CD3, CD4, CD8, CD14, CD16, CD20, CD45, and CD56, and with the specific CD20-CAR detection reagent, all provided by Miltenyi Biotec. Leukocytes were defined as CD45+ cells. All other leukocytes subpopulations were defined among the viable CD45+ cells (CD45+ / 7−AAD−) as follows: CD3+ cells (CD45+ / 7−AAD− / CD3+), NKT (CD45+ / 7−AAD− / CD3+ / CD16+ or CD56+), CD4+ cells (CD45+ / 7−AAD− / CD3+ / CD4+), CD8+ cells (CD45+ / 7−AAD− / CD3+ / CD8+), monocytes (CD45+ / 7−AAD− / CD14+), B cells (CD45+ / 7−AAD− / CD20+), and NK cells (CD45+ / 7−AAD− / CD16+ or CD56+). The identity of the CD20 CAR transduced cells was confirmed by the binding to the CD20 CAR-specific detection reagent, and the transduction frequency was measured as percentage of the CD20 CAR+ cell among CD3+ cells (CD45+ / 7−AAD− / CD3+ / CAR+). The proportion of CAR+ / CD4+ cells and CAR+ / CD8+ cells and the CAR+ / CD4+ / CD8+ (double positive) and the CAR+ / CD4− / CD8− (double negative) cells was additionally reported for information only.

Anti-CD62L and anti-CD45RO antibodies (Miltenyi Biotec) were used in a lyse/wash staining protocol for extended phenotyping analysis (complementary research). In addition to the described back-bone gating strategy for the determination of CD8+ and CD4+ cells further, T-cell populations were defined as follows: naive (N) T cells defined as CD62L+ / CD45RO−, central memory (CM) T cells defined as CD62L+ / CD45RO+, effector (E) T cells defined as CD62L− / CD45RO−, and effector memory (EM) defined as CD62L− / CD45RO+. These analyses were performed as complementary research only where the patient’s consent for this purpose was given.

#### Isolation of genomic DNA and determination of the vector copy numbers by qPCR

Genomic DNA was isolated from 1×10^6^ CD3+ cells using the DNeasy Blood & Tissue kit (Qiagen, Hilden, Germany). Reagents and materials were used as specified in the Qiagen RNeasy Blood & Tissue Kit manual. All DNA isolation steps were performed in a GMP-compliant environment. The isolated DNA was stored at −80°C (if necessary transported on dry ice) until further processing at Miltenyi Biotec. Determination of vector copy numbers (VCN) in CD20 CAR-transduced T cells was based on a duplex qPCR established at Miltenyi Biotec that quantified an integrated viral vector sequence fragment (gag) and normalized by the quantification of the cellular reference and single-copy gene PTBP2.

#### Microbiology

Microbiological examination according to chapter 2.6.27 of the European Pharmacopoeia (Ph. Eur. 2.6.27) was performed using the BD BACTEC blood culture system. For this purpose, 1% (v/v) of the IMP (but not <1 mL) was inoculated in BD BACTEC aerobic and anaerobic culture media (BD BACTEC™ Plus Aerobic/F- and BD BACTEC™ Plus Anaerobic/F- Medium, Becton, Dickinson and Company, Franklin Lakes, NJ, USA) and incubated automatically for 14 days.

#### Endotoxin

The presence of bacterial endotoxin was tested by a chromogenic kinetic method (Method D Ph. Eur. 2.6.14) using the Endosafe® nexgen-PTS™ device and FDA-licensed Endosafe® LAL cartridges (Charles River Laboratories International, Inc., Wilmington, MA, USA).

#### Mycoplasma

Nucleic acid amplification technique (NAT) was used as rapid test for mycoplasmas according to Ph. Eur. 2.6.7. Samples were drawn from the cell suspension, diluted to the maximal valid cell concentration, and sent on dry ice to the test laboratory (Minerva Analytix GmbH, Rangsdorf, Germany). The tests were completed within 24 h after sampling.

### *In vitro* functional testing

#### Cells and reagents for functional testing

Aliquots of IMP were cryopreserved with 10% dimethylsulfoxid and used later for extended characterization and functional testing, where patient’s consent for complementary research was given. Peripheral blood mononuclear cells (PBMCs) were purified from blood from healthy donors upon informed consent and approval by the institutional review board (21-2224-101 Regensburg) using density centrifugation on Pancoll human (PAN-Biotech, Aidenbach, Germany). JeKo-1 cells were purchased from ATCC (Manassas, VA, USA). Nalm6-GFP/Luc cells were obtained from T. Fry (National Cancer Institute, Bethesda, MD, USA). All cells were cultured in RPMI 1640 medium supplemented with GlutaMAX™ (Gibco/ThermoFisher, Waltham, MA, USA), 100 U/mL penicillin, 0.1 mg/mL streptomycin (PAN-Biotech), 0.01 M HEPES (Sigma-Aldrich, St. Louis, MO, USA), and 10% (v/v) FBS Good (PAN-Biotech), referred to as T-cell medium.

#### Activation of CAR T cells

CAR T cells (3×10^4^ T cells/well) were incubated in 96-well microwell plates coated with the agonistic anti-CD3 mAb OKT3 (1 μg/mL, BioLegend, San Diego, CA, USA) and anti-CD28 mAb CD28.2 (5 μg/mL, BioLegend) or PBS for control. After 48 h, supernatants were tested for IFN-γ by ELISA using solid phase bound anti-IFN-γ capture and biotinylated anti-IFN-γ detection antibody (BD Bioscience, Franklin Lakes, NJ, USA). The reaction product was visualized with a peroxidase–streptavidin conjugate (1:10,000) and ABTS (Roche Diagnostics, Mannheim, Germany). Alternatively, CAR T cells (1×10^5^ total T cells/well) were co-cultivated for 72 h in 96-well microwell plates with tumor cells (1×10^5^ cells/well), with the agonistic anti-CD3 mAb OKT3 (1 μg/mL, BioLegend) and anti-CD28 mAb CD28.2 (5 μg/mL, BioLegend) or PBS for control. After 72 h, supernatants were harvested and stored at −20°C. After thawing, supernatants were diluted 1:2 with Calibrator Diluent RD6-52, and a 11-plex human Th1/Th2 cytokine panel was used for determination of granulocyte-macrophage colony-stimulating factor (GM-CSF), interleukin (IL)-1 β/IL-1F2, IL-4, IL-6, IL-13, TNF-α, IFN-γ, IL-2, IL-5, IL-12 p70, and IL-18/IL-1F4 on the Luminex® platform. The Luminex® Discovery Assay (R&D Systems, Minneapolis, MN, USA) was performed according to the manufacturer’s manual utilizing the Bio-Plex MAGPIX Multiplex Reader and the software xPONENT for MAGPIX 4.2 (Luminex Corporation, Austin, TX, USA).

#### Degranulation assay

GFP-labeled Nalm6 and JeKo-1 cells were seeded in 12-well plates at 1×10^5^ cells per well, and 1×10^5^ CAR T cells were added per well (see round 1, “stress test”). CAR T cells were stained for CD107a expression after round 1 of antigen stimulation. For flow analysis, a respective gate was set, and the mean fluorescence (MFI) of CD107a staining after co-incubation with Nalm6 was compared to the MFI of CD107a expression after co-incubation with JeKo-1 cells.

#### Repetitive stimulation assay (“stress test”)

GFP-labeled Nalm6 and JeKo-1 cells were seeded in 12-well plates at 1×10^5^ cells per well, and 1×10^5^ CAR T cells were added per well. After 3 days (round 1, R1), all cells were harvested and resuspended in 1 mL T-cell medium. Finally, 0.1 mL was used for cell counting (live GFP+ cancer cells and live CD3+/CAR+ CAR T cells) by flow cytometry using “AccuCheck Counting Beads” (Thermo Fisher Scientific, Waltham, MA, USA), and the remaining 0.9 mL was added to a new 12-well plate with fresh 1×10^5^ CD20^low^ Nalm6 and CD20^high^ JeKo-1 cells for 4 days (round 2, R2). The procedure was re-iterated for round 3 (R3) and round 4 (R4).

#### Flow cytometry

The viability dye “eFluor 780” (Thermo Fisher Scientific) was used to exclude dead cells from analysis. CARs were detected using the PE-labeled “CD20 detection reagent” (Miltenyi Biotec). The following antibodies were purchased from BioLegend: BV421-labeled anti-CD3 antibody to identify T cells, PE-labeled CD20 antibody, PE-labeled mouse IgG2b, Igκ isotype control antibody, and APC-labeled CD107a antibody. Flow cytometry was performed using a FACSLyric™ flow cytometer equipped with FACSuite™ v1.5 software or a FACSymphony™ flow cytometer equipped with FACSDiva™ v9.0 software (BD Bioscience).

### Statistical analyses

Descriptive statistics and statistical significance (by two-tailed paired or unpaired t test, alpha=0.05) were calculated using GraphPad Prism 9.5.1.733 or 10.1.0 (GraphPad Software LLC, Boston, MA USA).

## Results

### Automated production of CAR+ T cells using CliniMACS Prodigy^®^

**Figure 1:**
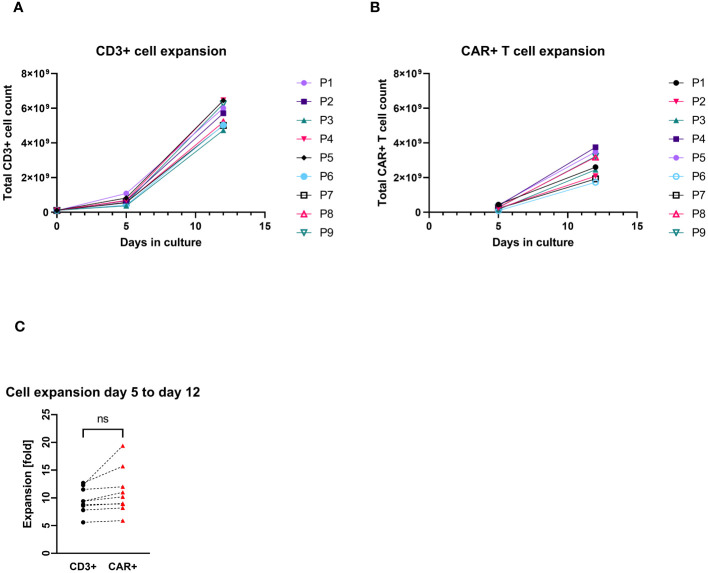
Total cell count expansion during the cell culturing (P1–P9 = patient numbers). **(A)** CD3+ cell expansion with cell counts measured at day 0 (at start culturing), day 5, and day 12 (harvest). **(B)** CAR+ T-cell expansion with cell counts measured at day 5 (4 days after transduction) and day 12 (harvest). **(C)** Pairwise comparison of expansion rates of CD3+ and CAR+ T cells (each connecting line corresponds to an individual production process); “ns”, not significant difference between the groups.

All patients’ apheresis products delivered sufficient CD3+ cell counts for the manufacturing process. CD4+ and CD8+ cells were successfully enriched to a purity of the target fraction ≥83%. The median recovery of the sum of CD4+ and CD8+ cells was 82% (max/min, 70%–37%). The transduction and cell cultivation started with approximately 1×10^8^ CD3+ cells, independent of the final dose required. The median expansion of CD3+ cells was 53-fold (max/min, 65–42-fold). The median transduction rate was at day 12 was 52% CAR+ cells among CD3+ cells (max/min, 61%–34%), which allowed harvesting of median 2.6×10^9^ CAR+ T cells (max/min, 3.8–1.7×10^9^). There was no significant difference between the expansion rate of the general CD3+ cell population and the CAR+ T cells quantified at day 5 and day 12 of the production process (**Figure 1**). All harvested products fulfilled the requirements even for the largest clinical dose (1×10^7^/kg patient’s body weight) as planed in the study protocol. The viability of CD3+ cells in one of the IMPs after formulation was less than the limit of 80% (79.9%), which resulted in an out of specification event (OOS). Another product resulted also in OOS concerning appearance. Small aggregates were visible after formulation of the cell suspension. These products were not released but applied outside the clinical trial with the patients’ consent and according to a benefit risk assessment by the investigator.

### Cell composition

**Figure 2:**
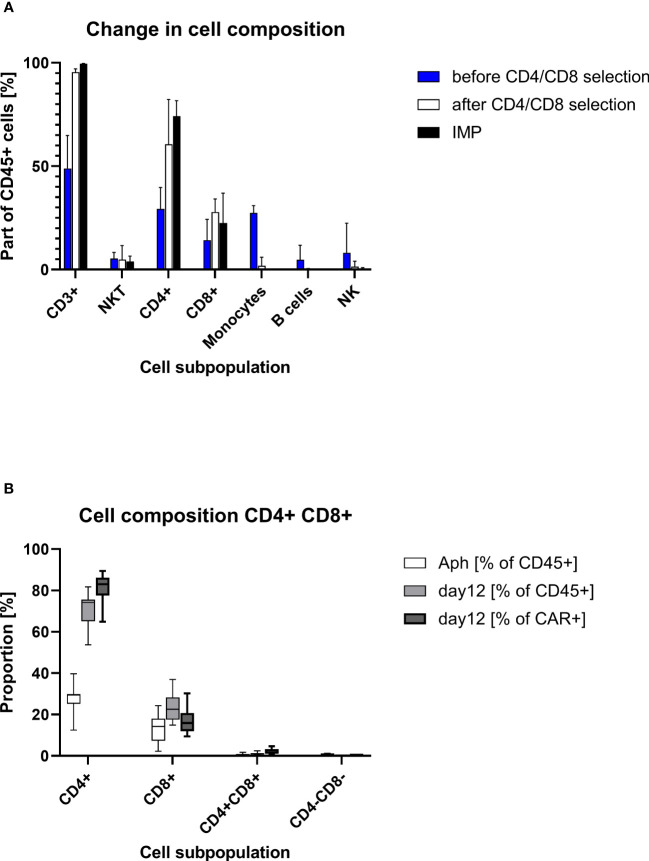
Cell composition on different points of the production process. **(A)** Descriptive column chart (median with range) of cell composition before and after CD4/CD8 T-cell selection and at the end of cell culturing at day 12 (IMP). **(B)** Descriptive standard box plot diagram of proportions of CD4+ and CD8+ cells at start (apheresis) and in the end of the production process (day 12). The whiskers indicate the smallest and largest value.

Starting with approximately 50% CD3+ cells in the apheresis (median/max/min, 48.9%/64.9%/17.1%), the proportion of CD3+ cells was considerable greater after CD4/CD8 combined selection (med/max/min, 95.5%/97.0%/89.3%). The percentage of CD3+ cells at the end of the cell culturing was in the median of 99.5% (max/min, 99.7%/98.5%). A median of 74.2% (max/min, 81.7/53.7) of the CD45+ cells in the final product was CD4+. The proportion of CD3+CD8+ cell comprised in median 22.5% (max/min, 36.9%/15.0%) of the CD45+ cells. While very low amounts of B cells (med/max/min, 0.2%/0.5%/0.03%), monocytes (med/max/min, 1.9%/6.0%/0.2%), and NK (med/max/min, 1.5%/4.1%/0.9%) cells were still contaminating the target cell fraction after CD4+/CD8+ selection, no monocytes were detected at the end of culturing (in the IMP). Negligible amounts of B cells (0.01% at detection limit) were measured in one IMP. The proportion of NK cells was reduced to a median of 0.06% (max/min, 0.95%/0.01%) in the IMP (**Table 2**, **Figure 2**).

**Table 2:** Cell composition IMP.

		P1	P2	P3	P4	P5	P6	P7	P8	P9	median
% of CD45+	CD3+	99.6	99.5	98.5	99.7	99.5	99.5	99.5	99.5	99.5	99.5
	NKT	1.6	2.1	3.8	3.9	2.6	6.2	6.0	6.6	4.5	3.9
	CD4+	74.2	81.7	68.4	68.0	75.4	62.2	53.7	75.8	75.2	74.2
	CD8+	22.5	14.9	24.9	27.2	20.8	29.5	36.9	16.1	19.0	22.5
	Monocytes	0.0	0.0	0.0	0.0	0.0	0.0	0.0	0.0	0.0	0.0
	B cells	0.0	0.0	0.0	0.0	0.0	0.0	0.0	0.0	0.0	0.0
	CD56+ CD16+ cells	0.1	0.1	1.0	0.0	0.1	0.2	0.2	0.1	0.1	0.1
	CAR+ cells (% of CD3+)	43.3	36.4	51.9	58.1	53.9	34.4	38.3	61.2	51.5	51.5
% of CAR+	CAR+CD4+	86.5	89.4	86.0	79.4	82.2	75.7	64.9	83.2	83.1	83.1
	CAR+CD8+	11.7	9.4	12.4	19.5	16.6	22.0	30.2	12.2	15.9	15.9
	CAR+CD4+ CD8+	1.7	1.1	1.5	1.1	1.2	2.2	4.6	4.5	1.0	1.5
	CAR+ CD4- CD8-	0.1	0.1	0.1	0.1	0.1	0.2	0.3	0.1	0.1	0.1

**Figure 3:**
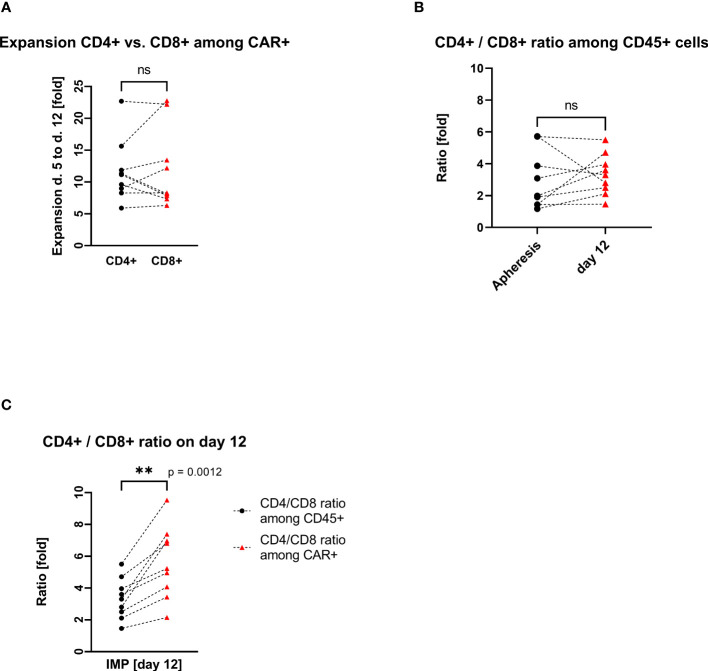
CD4+ and CD8+ T-cell expansion and ratio (each connecting line corresponds to an individual production process); “ns”, not significant difference between the groups. **(A)** Pairwise comparison of CD4+ and CD8+ T-cell expansion during cell culturing. **(B)** Pairwise comparison of CD4+/CD8+ cell ratio among CD45+ cells at the start (apheresis) and at the end (day12) of the manufacturing process. **(C)** Pairwise comparison of CD4+/CD8+ cell ratio among CD45+ cells vs. CD4+/CD8+ cell ratio among CAR+ T cells on day 12. ** statistically significant difference.

The transduced CD4+ and CD8+ cells showed a similar expansion pattern (cell count measured at day 5 and day 12). The ratio CD4+/CD8+ in the IMP was not significantly different from the initial CD4+/CD8+ ratio in the corresponding apheresis. Interestingly, the percentage of CD4+ cells among the CAR+ T-cell population (med/max/min, 83.1%/89.4%/64.9%) was higher than in the general CD45+ cell population. Respectively, the percentage of CD8+ cells was lower among the CAR+ T-cell population (med/max/min, 15.9%/30.2%/9.4%) than within the general CD45+ cell population. This shift is be represented by the ratio CD4+/CD8+, which differs significantly (p=0.0012) among CD45+ cells (med/max/min, 3.3/5.5/1.5-fold) in comparison with the ratio among CAR+ T cells (med/max/min, 5.2/9.5/2.2-fold) (**Figure 3**).

Advanced flow cytometry for T-cell subpopulations was performed as complementary research only in case of given patient’s consent for this type of analysis. The tested samples pointed out mostly a central memory phenotype of the produced CAR T cells as defined by CD62L and CD45RO expression (exemplary shown on **Supplementary Figure S1B**). Less than 10% of the CAR+ cells at harvest were defined as effector memory T cells. The proportions of naive and effector CAR+ T cells were <1% at harvest. For comparison, at start of the cell cultivation (after CD4+/CD8+ T cell selection), all T-cell subpopulations are presented as shown exemplary on **Supplementary Figure S1A**.

### VCN, microbiological examination, endotoxin, and mycoplasma

A median of 1.47 (n=8; max/min, 2.1/1.1) vector copy numbers per transduced cell was determined in the IMP. Therewith, the specification for VCN being <5 copies per transduced cell was met for all manufactured IMPs. Similarly, all manufactured batches complied with the product specifications (IPC and QC) for microbiological examination, endotoxin, and mycoplasma (**Table 1**).

### Manufacturing at two production sites

**Figure 4:**
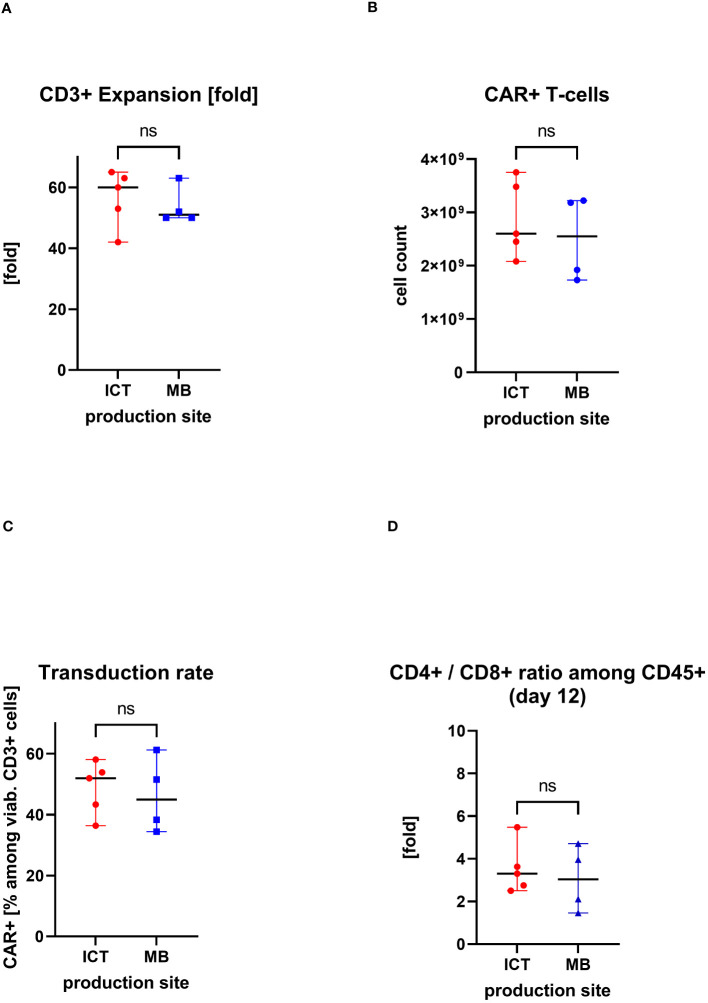
Comparison of two production sites (individual values with median, range); ns, statistically not significant. **(A)** Cell expansion after 12 days of culturing. **(B)** Obtained total count of CAR+ T cells at the end of the manufacturing process. **(C)** Proportion of CAR+ T cells in the final CAR T-cell product (transduction rate). **(D)** CD4+/CD8+ ratio in the final CAR T cell product.

IMPs were produced at two manufacturing sites, at the Institute of Cellular Therapeutics (ICT, department Cellular Therapy Centre), Hannover Medical School (n=5) and at Miltenyi Biotec (MB) (n=4). There were no significant differences in the obtained cell products between the production sites. The median expansion rate of CD3+ cells throughout 12 days cell culturing was at ICT of 60-fold (max/min, 65–42-fold) versus median of 51-fold (max/min, 63/50-fold) at MB. The median transduction rate at ICT was 52% (max/min, 58%/36%) versus median of 45% (max/min, 61%/34%) at MB. This led to a total CAR+ T-cell count of median of 2.6×10^9^ at ICT (max/min, 3.8/2.1×10^9^) versus median of 2.6×10^9^ (max/min, 3.2/1.7×10^9^) at MB. Also similar was the ratio of CD4+/CD8+ cells in the IMP: median of 3.3-fold (max/min, 5.5/2.5-fold) at ICT versus median of 3.0 (max/min, 4.7/1.5-fold) at MB (**Figure 4**).

### Functional capacities of manufactured CAR T-cell products

**Figure 5:**
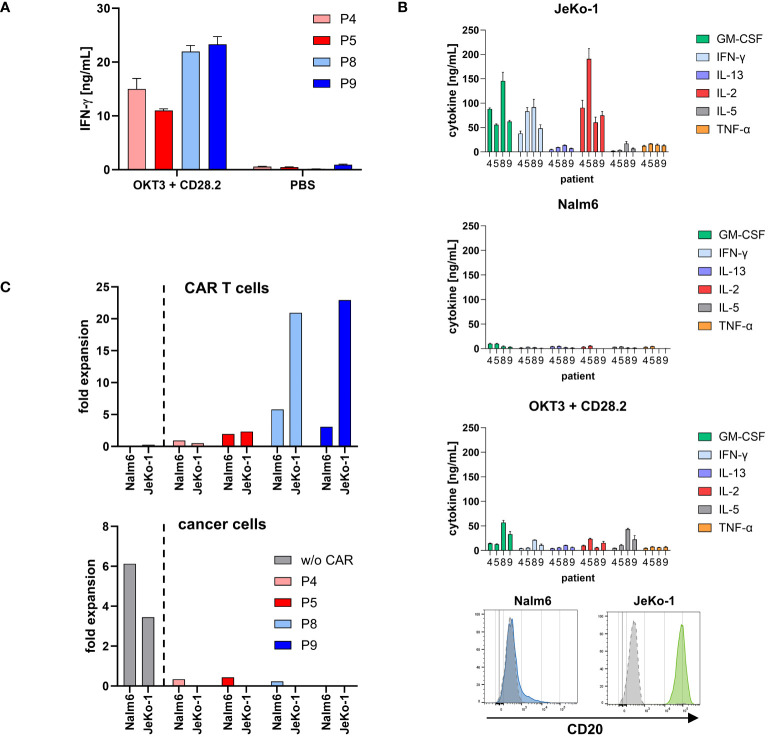
CD20 CAR T cells from both production sites were specifically activated. **(A)** Cell products were functionally activated by stimulation through TCR/CD28. Microtiter plates were coated with the agonistic anti-CD3 mAb OKT3 (1 μg/mL) and anti-CD28 mAb CD28.2 (5 μg/mL) or PBS for control. Cell products (3×10^4^ total T cells/well) were incubated for 48 h. IFN-γ in the supernatant was determined by ELISA. Data represent mean values of technical triplicates ± SD. One out of at least three experiments is shown. **(B)** CD20 CAR T cells secreted a panel of cytokines upon stimulation with CD20 positive cancer cells. CAR T cells (1×10^5^ total T cells) were co-incubated with 1×10^5^ CD20^low^ Nalm6 cells or CD20^high^ JeKo-1 cells, respectively, for 72 h. Alternatively, microtiter plates were coated with the agonistic anti-CD3 mAb OKT3 (1 μg/mL) and anti-CD28 mAb CD28.2 (5 μg/mL) or PBS for control. CAR T cells (1×10^5^ total T cells/well) were incubated for 72 h. Cytokines in the supernatant were determined by Luminex® Discovery Assay. Data represent mean values of technical triplicates ± SD. Test results of IL-1 β/IL-1F2, IL-4, IL-6, IL-12 p70, and IL-18/IL-1F4, and test results of CAR T cells without stimulation (PBS control) are below 0.5 ng/mL and are not shown. CD20 expression of the target cell lines Nalm6 (blue histogram) and JeKo-1 (green histogram) upon staining with a CD20-PE antibody or isotype-matched control antibody (gray histograms) is shown as recorded by flow cytometry. **(C)** Expansion of CD20 CAR T cells was calculated by dividing the absolute number of CAR T cells after first round of antigen stimulation with CD20^low^ Nalm6 cells or CD20^high^ JeKo-1 cells by the respective number on day 0. Data were extracted from **Figure 6B**. Non-modified T cells of a healthy donor (w/o CAR) served as control. The number of cancer cells was likewise calculated by dividing the absolute number of cancer cells after first round of co-incubation with non-modified T cells or CAR T cells by the respective number on day 0.

Cryopreserved portions of two cell products from each manufacturing site were chosen for functional testing in detail as complementary research. As shown in **Figure 5A**, all cell products were functionally activated by stimulation through the TCR/CD28 as indicated by an increase in IFN-γ release; only background IFN-γ release was recorded without stimulation. As expected, there were differences in the degree of activation upon TCR/CD28 stimulation between the patients.

To monitor the functional capacities upon CAR engagement of target cells, CD20 CAR T cells were incubated with CD20^high^ JeKo-1 and CD20^low^ Nalm6 cells, respectively. CAR engagement of JeKo-1 cells induced substantial cytokine release by all CAR T-cell products, while engagement of CD20^low^ Nalm6 cells induced low amounts of cytokines, however, above background levels (**Figure 5B**). Again, all cell products were functionally activated by stimulation through the TCR/CD28 as indicated by pro-inflammatory cytokine release. Of note, stimulation through high levels of CD20 on target cells more potently induced cytokine release.

CAR T cells started to proliferate upon recognition of CD20+ target cells as recorded by flow cytometry after 3 days of stimulation (**Figure 5C**). Higher CAR T-cell amplification was recorded upon engagement of CD20^high^ JeKo-1 cells compared to engagement of CD20^low^ Nalm6 cells throughout all cell products. Again, the degree in CAR T-cell amplification was different between the individual patients. Non-modified T cells of a healthy donor showed no proliferative capacity. In line with these findings, Nalm6 and JeKo-1 cells proliferated when co-incubated with non-modified T cells, whereas there was no amplification of cancer cells in coculture with CAR T cells.

**Figure 6:**
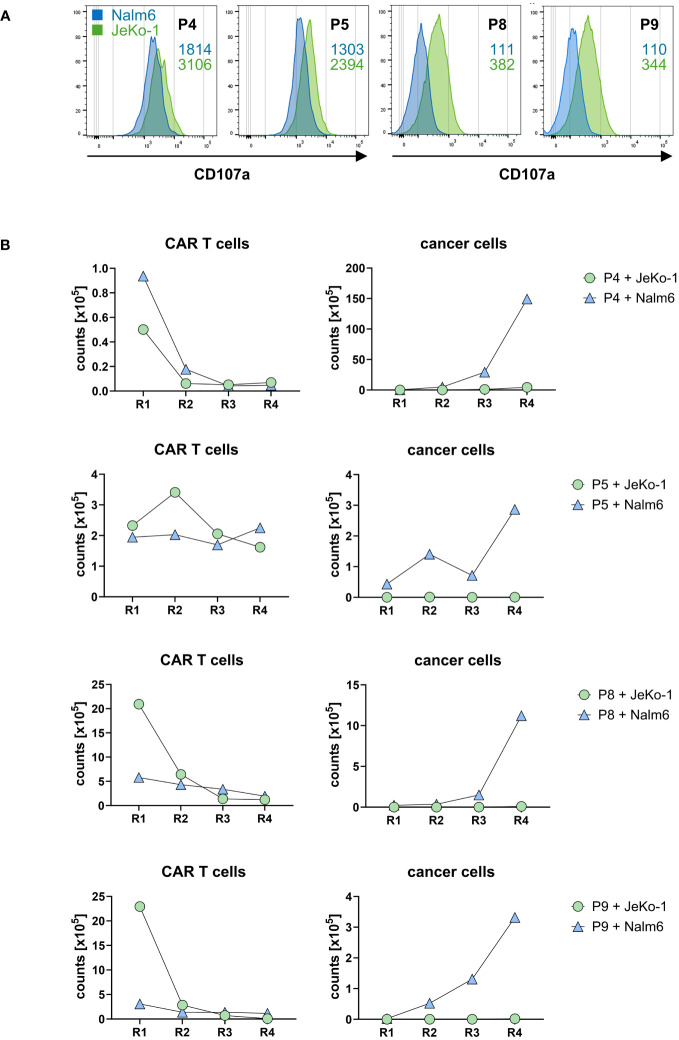
CD20 CAR T cells exhibited cytolytic capacities. **(A)** CAR T cells were stained for CD107a after first round of antigen stimulation with CD20^low^ Nalm6 cells or CD20^high^ JeKo-1 cells as described in panel **(B)**. CD107a was detected by flow cytometry, and MFI values upon co-incubation with Nalm6 cells (blue numbers) or JeKo-1 cells (green numbers) are indicated. The samples were analyzed in two batches, i.e., batch 1 (P4, P5) and batch 2 (P8, P9). **(B)** CD20 CAR T cells eliminate CD20-positive cancer cells upon repetitive stimulation. CAR T cells (starting with 1×10^5^ CAR T cells) were subjected to four rounds (R1–R4) of stimulation with GFP-labeled CD20^low^ Nalm6 cells or CD20^high^ JeKo-1 cells (1×10^5^ cancer cells at start of each round). At the end of each round, CAR T cells (live CD3+/CAR+) and cancer cells (live GFP+ Nalm6 or JeKo-1 cells) were counted by flow cytometry using counting beads.

Co-incubation with CD20+ target cells induced a cytolytic CAR T-cell response indicated by the induction of CD107a upon lytic degranulation. CD20^high^ JeKo-1 cells induced higher lytic degranulation of CAR T cells compared to CD20^low^ Nalm6 cells (**Figure 6A**), which is in accordance with the higher degree in cytokine activation upon JeKo-1 compared to Nalm6 cell co-incubation (cf. **Figure 5B**).

To read the CAR T-cell cytolytic capacities under conditions of repetitive rounds of antigen stimulation (“stress test”), CD20 CAR T cells starting with 10^5^ cells were co-incubated with the same number of GFP-marked JeKo-1 or Nalm6 cells, respectively, and both CAR T cells and cancer cells were counted by flow cytometry after a stimulation period of 3 days. A defined number of fresh JeKo-1 and Nalm6 cells, respectively, were added to the cells after this first round and co-incubated for a second period. The procedure was re-iterated for rounds 3 and 4. As summarized in **Figure 6B**, CD20^high^ JeKo-1 cells were nearly completely eliminated in the first and all following rounds of re-stimulation. CD20^low^ Nalm6 cells were initially also eliminated, however, increasingly persisted in rounds 3 and round 4, indicating a declining efficacy of the lytic CAR T-cell attack towards CD20^low^ cells, whereas the CAR T cells were still efficient in eliminating CD20^high^ cells. This is substantiated by declining CAR T-cell numbers in rounds 3 and 4, whereas in round 1, the CAR T cells vigorously amplified. The same observations were made basically for all four CAR T-cell products tested. Taken together, all cell products were capable to recognize CD20+ target cells, to become activated as indicated by CAR T-cell expansion and increase in IFN-γ release and to eliminate CD20+ cancer cells under conditions of repetitive target cell encounter.

## Discussion

Decentralized point-of-care (POC) manufacturing of CAR T cells using the automated CliniMACS Prodigy® platform is increasingly applied worldwide (28). The decentralization of manufacturing offers reduced production time due to lack of complex transport logistic and the possibility of delivery of CAR T-cell products without cryopreservation, which is of particular relevance for at least early phase I/II clinical trials. In addition, the automated CAR T-cell process gives rise for a cost-balanced scalable use all around the world as shown by Palani et al. in India (29).

One of the prerequisites for decentralized POC is the automatization of the production process allowing standardization of the manufacturing with similar results at different production sites. As a recent example of one of the largest trials, Maschan et al. reported on CAR T cells redirected against CD19 for ALL (n=31) and NHL (n=23) patients, whereby the manufacturing was performed on two different sites (30). The overall purity of the final product reached >90% CD3+ T cells with similar cell composition and transduction efficiency at both manufacturing sites (60% and 48%, respectively). Similarly, we report here data (however on CD20 CAR T cells) from two production sites, with no significant differences regarding the cell expansion rate (median 60- versus 51-fold), the amount of produced CAR T cells, CD4+/CD8+ ratio, and even closer transduction efficiency being in median of 52% and 45%, respectively. The better comparability between both production sites in our study may be due to the greater consistency of the starting material, i.e., the leukapheresis from patients with the same diagnosis.

To date, the CliniMACS Prodigy® has been mostly used for the manufacturing of anti-CD19 CAR T cells. In contrast to that, limited results have been published so far about manufacturing of CAR T cells redirected against other cancer epitopes. Due to the clinical need to achieve a long-term effect against CD20+ melanoma stem cells, which could not been reached with Rituximab alone, we established a manufacturing process for anti-CD20 CAR T cells on the CliniMACS Prodigy®. As we showed previously by validation runs, CD4+/CD8+ enrichment followed by transduction with a second generation CAR equipped with 4-1BB-CD3ζ signaling chains led to a robust cell product (23). Starting with leukapheresis material from healthy donors during the validation, we reached a median purity of 97% T cells with a median of 65-fold cell expansion and overall 23% transduction efficiency, which was significantly higher for CD4+ compared to CD8+ cells. The validated production protocol was the basis for starting the clinical trial in stage III/IV melanoma patients (NCT03893019) using parallel production of CD20+ CAR T cells at two different manufacturing sites. A high expansion rate of 53-fold (median from nine production runs) was reached also with autologous cells from the stage III/IV melanoma patients, which confirmed the suitability of the manufacturing process for clinical application.

Interestingly, the transduction frequency (median, 52%) was higher compared to the five validation runs starting with leukapheresis products taken from healthy donors (23%). The higher transduction efficiency compensated the a bit lower general T-cell expansion, which resulted in comparable CAR T-cell yield at harvest (median of 1.5×10^9^ in the validation runs vs. median of 2.6×10^9^ CAR T cells in the clinical runs). The superior transduction efficiency of CD4+ cells in comparison to the CD8+ cells obtained during the validation runs was confirmed in the clinical trial production runs. The CD4+/CD8+ ratio was in the median of 3.3-fold, when the entire CD45+ cell population was analyzed and median of 5.2-fold among the CAR+ T cells only.

In a detailed analysis, we monitored the functional capacities of the manufactured CAR T cells from both production sites in a comparative setting. All tested CAR T-cell products were capable to translate into T-cell activation upon engagement of CAR target cells, indicated by the increase in IFN-γ as a lead cytokine and by increase in CAR T-cell amplification. In line therewith, all T-cell products were susceptible to stimulation through their endogenous TCR/CD28. Notably, there were some inter-individual, cell-intrinsic differences at the level of cytokine release and amplification that became likewise obvious through TCR/CD28 and CAR stimulation.

CAR-mediated T-cell activation depended on the level of CAR cognate antigen, as JeKo-1 cells with high CD20 levels evoked a stronger response than Nalm6 cells with low CD20 levels. This has functional consequences, as upon several rounds of re-stimulation with the respective target cells, CD20^low^ Nalm6 cells were less efficiently eliminated than CD20^high^ JeKo-1 cells. The observation holds for all four tested cell products from the two production sites pointing to a more general, production-site-independent feature of antigen dose on CAR-mediated T-cell activation.

Due to the small patients’ pool in our study and to the fact that complementary research could be done only with given patient’s consent, limited analysis on the advanced cell phenotyping was conducted. The tested samples pointed out mostly central memory phenotype of the produced anti-CD20 CAR T cells, which corresponds to other CAR T cells with 4-1BB signaling domain as summarized by Niels Schaft (31). This is also in line with the observation during the manufacturing process of anti-CD19 CAR T cells in a large cohort as described by Teoh and Brown (32). They described a shift in the memory T-cell phenotypic composition from the starting material to the final CAR T-cell product for patients with large B-cell lymphoma, mantle cell lymphoma, or chronic lymphocytic leukemia. However, they also observed direct correlation between the differentiation state of the starting material (patient apheresis samples and selected T-cell material) and the produced CAR T cells. This observation demonstrates the influence of the autologous starting material on the product quality and supports the need of larger patients/products pool for being able to perform deeper analysis on that attributes.

Beside substantial progress in automatization of the CAR T-cell manufacturing, efforts have also been made to shorten the process through an expansionless “next day” manufacturing (33–35). This has been also reported for the commercial product “*tisagenlecleucel*”, where manufacturing switched to a novel T-Charge™ platform in a 2-day process, which preserves T-cell stemness (36). Although these automated and optimized protocols are important steps towards improved manufacturing, more advanced technologies are needed to enable the treatment of large patient groups, especially if treatment reaches diseases beyond cancer (37).

## Conclusion

The reported results on manufacturing of clinical-grade well-characterized fresh CAR T cells as investigational medicinal product demonstrated that the point-of-care platform is well suited for decentralized automated CAR T-cell manufacturing saving valuable patient treatment time. This was shown for anti-CD20 CAR T cells and will most likely also apply to targeting other cancer epitopes or giving rise for further applications beyond cancer similar to the current use of anti-CD19 CAR for treatment of systemic lupus erythematosus (38, 39) and the anti-synthetase syndrome (40).

## Data availability statement

The original contributions presented in the study are included in the article/**Supplementary Material**. Further inquiries can be directed to the corresponding authors.

## Ethics statement

The studies involving humans were approved by Ethikkommission der Medizinischen Fakultät, Universität zu Köln, Cologne, Germany, the Ethikkommission der Medizinischen Fakultät, Ludwig-Maximilians-Universität, Munich, Germany and by the institutional review board of the University of Regensburg (21-2224-101 Regensburg), Regensburg, Germany. The studies were conducted in accordance with the local legislation and institutional requirements. The participants provided their written informed consent to participate in this study.

## Author contributions

KA: Data curation, Formal analysis, Investigation, Methodology, Validation, Visualization, Writing – original draft. JL: Data curation, Formal analysis, Investigation, Methodology, Validation, Visualization, Writing – original draft. CP: Writing – review & editing, Data curation, Investigation, Methodology. MAk: Conceptualization, Project administration, Writing – review & editing, Supervision. MAp: Writing – review & editing, Conceptualization, Project administration, Resources, Writing – original draft. MAs: Investigation, Writing – review & editing. IB: Writing – review & editing, Conceptualization, Investigation. AR: Writing – review & editing, Investigation. PA: Writing – review & editing, Project administration. CS: Writing – review & editing, Project administration. AH: Data curation, Investigation, Writing – original draft, Writing – review & editing. MB: Data curation, Investigation, Writing – original draft, Writing – review & editing. VB: Data curation, Investigation, Writing – original draft, Writing – review & editing. Mv: Investigation, Writing – review & editing. PB: Investigation, Writing – review & editing. LG: Investigation, Writing – review & editing. WG: Investigation, Methodology, Writing – original draft, Writing – review & editing, Data curation. LA: Conceptualization, Investigation, Methodology, Project administration, Supervision, Writing – original draft, Writing – review & editing, Validation. RE: Investigation, Methodology, Writing – review & editing, Formal analysis, Validation, Writing – original draft. HA: Investigation, Writing – review & editing, Data curation, Writing – original draft. UK: Conceptualization, Investigation, Writing – review & editing, Formal analysis, Funding acquisition, Methodology, Project administration, Resources, Supervision, Writing – original draft.

## Supplementary material

Supplementary Figure 1 Exemplary flow cytometric dot plots on phenotyping of CD8+ and CD4+ cells using CD62L (y-axis) and CD45RO (x-axis) expression. Naïve (N) T cells defined as CD62L+CD45RO-, Central memory (CM) T cells defined as CD62L+CD45RO+, Effector (E) T cells defined as CD62L-CD45RO- and effector memory (EM) defined as CD62L-CD45RO+ are shown on dot plots, gated on CD3+CD8+ (left) or CD3+CD4+ (right) respectively. **(A)** T cells after CD4/CD8 T cell selection and **(B)** CAR+ T cells at harvest.

## References

[1] Chapman PB Hauschild A Robert C Haanen JB Ascierto P Larkin J. Improved survival with vemurafenib in melanoma with BRAF V600E mutation. N Engl J Med. (2011) 364:2507–16. doi: 10.1056/NEJMoa1103782 PMC3549296 21639808

[2] Hauschild A Grob JJ Demidov LV Jouary T Gutzmer R Millward M. Dabrafenib in BRAF-mutated metastatic melanoma: a multicentre, open-label, phase 3 randomised controlled trial. Lancet. (2012) 380:358–65. doi: 10.1016/S0140-6736(12)60868-X 22735384

[3] Robert C Long GV Brady B Dutriaux C Maio M Mortier L. Nivolumab in previously untreated melanoma without BRAF mutation. N Engl J Med. (2015) 372:320–30. doi: 10.1056/NEJMoa1412082 25399552

[4] Weber JS D'Angelo SP Minor D Hodi FS Gutzmer R Neyns B. Nivolumab versus chemotherapy in patients with advanced melanoma who progressed after anti-CTLA-4 treatment (CheckMate 037): a randomised, controlled, open-label, phase 3 trial. Lancet Oncol. (2015) 16:375–84. doi: 10.1016/S1470-2045(15)70076-8 25795410

[5] Schachter J Ribas A Long GV Arance A Grob JJ Mortier L. Pembrolizumab versus ipilimumab for advanced melanoma: final overall survival results of a multicentre, randomised, open-label phase 3 study (KEYNOTE-006). Lancet. (2017) 390:1853–62. doi: 10.1016/S0140-6736(17)31601-X 28822576

[6] Wolchok JD Chiarion-Sileni V Gonzalez R Rutkowski P Grob JJ Cowey CL. Overall survival with combined nivolumab and ipilimumab in advanced melanoma. N Engl J Med. (2017) 377:1345–56. doi: 10.1056/NEJMoa1709684 PMC5706778 28889792

[7] Larkin J Chiarion-Sileni V Gonzalez R Grob JJ Rutkowski P Lao CD. Five-year survival with combined nivolumab and ipilimumab in advanced melanoma. N Engl J Med. (2019) 381:1535–46. doi: 10.1056/NEJMoa1910836 31562797

[8] Sun W Xu Y Yan W Wang C Hu T Luo Z. A real-world study of adjuvant anti-PD -1 immunotherapy on stage III melanoma with BRAF, NRAS, and KIT mutations. Cancer Med. (2023) 12(15):15945–54. doi: 10.1002/cam4.6234 PMC10469738 37403699

[9] Schlaak M Schmidt P Bangard C Kurschat P Mauch C Abken H. Regression of metastatic melanoma in a patient by antibody targeting of cancer stem cells. Oncotarget. (2012) 3:22–30. doi: 10.18632/oncotarget.v3i1 22289880 PMC3292889

[10] Kalos M Levine BL Porter DL Katz S Grupp SA Bagg A. T cells with chimeric antigen receptors have potent antitumor effects and can establish memory in patients with advanced leukemia. Sci Transl Med. (2011) 3:95ra73. doi: 10.1126/scitranslmed.3002842 PMC3393096 21832238

[11] Porter DL Levine BL Kalos M Bagg A June CH. Chimeric antigen receptor-modified T cells in chronic lymphoid leukemia. N Engl J Med. (2011) 365:725–33. doi: 10.1056/NEJMoa1103849 PMC3387277 21830940

[12] Kochenderfer JN Dudley ME Carpenter RO Kassim SH Rose JJ Telford WG. Donor-derived CD19-targeted T cells cause regression of Malignancy persisting after allogeneic hematopoietic stem cell transplantation. Blood. (2013) 122:4129–39. doi: 10.1182/blood-2013-08-519413 PMC3862276 24055823

[13] Davila ML Riviere I Wang X Bartido S Park J Curran K. Efficacy and toxicity management of 19-28z CAR T cell therapy in B cell acute lymphoblastic leukemia. Sci Transl Med. (2014) 6:224ra25. doi: 10.1126/scitranslmed.3008226 PMC4684949 24553386

[14] Maude SL Frey N Shaw PA Aplenc R Barrett DM Bunin NJ. Chimeric antigen receptor T cells for sustained remissions in leukemia. N Engl J Med. (2014) 371:1507–17. doi: 10.1056/NEJMoa1407222 PMC4267531 25317870

[15] Lee DW Kochenderfer JN Stetler-Stevenson M Cui YK Delbrook C Feldman SA. T cells expressing CD19 chimeric antigen receptors for acute lymphoblastic leukaemia in children and young adults: a phase 1 dose-escalation trial. Lancet. (2015) 385:517–28. doi: 10.1016/S0140-6736(14)61403-3 PMC7065359 25319501

[16] Schmidt P Kopecky C Hombach A Zigrino P Mauch C Abken H. Eradication of melanomas by targeted elimination of a minor subset of tumor cells. Proc Natl Acad Sci U.S.A. (2011) 108:2474–9. doi: 10.1073/pnas.1009069108 PMC3038763 21282657

[17] Pinc A Somasundaram R Wagner C Hormann M Karanikas G Jalili A. Targeting CD20 in melanoma patients at high risk of disease recurrence. Mol Ther. (2012) 20:1056–62. doi: 10.1038/mt.2012.27 PMC3345981 22354376

[18] Zabierowski SE Herlyn M. Melanoma stem cells: the dark seed of melanoma. J Clin Oncol. (2008) 26:2890–4. doi: 10.1200/JCO.2007.15.5465 18539969

[19] Schmidt P Abken H. The beating heart of melanomas: a minor subset of cancer cells sustains tumor growth. Oncotarget. (2011) 2:313–20. doi: 10.18632/oncotarget.v2i4 PMC3248160 21487158

[20] Seftor EA Margaryan NV Seftor REB Hendrix MJC. Heterogeneity of melanoma with stem cell properties. Adv Exp Med Biol. (2019) 1139:105–14. doi: 10.1007/978-3-030-14366-4_6 31134497

[21] Cappell KM Kochenderfer JN. A comparison of chimeric antigen receptors containing CD28 versus 4-1BB costimulatory domains. Nat Rev Clin Oncol. (2021) 18:715–27. doi: 10.1038/s41571-021-00530-z 34230645

[22] Priesner C Aleksandrova K Esser R Mockel-Tenbrinck N Leise J Drechsel K. Automated enrichment, transduction, and expansion of clinical-scale CD62L(+) T cells for manufacturing of gene therapy medicinal products. Hum Gene Ther. (2016) 27:860–9. doi: 10.1089/hum.2016.091 PMC5035932 27562135

[23] Aleksandrova K Leise J Priesner C Melk A Kubaink F Abken H. Functionality and cell senescence of CD4/ CD8-selected CD20 CAR T cells manufactured using the automated cliniMACS prodigy(R) platform. Transfus Med Hemother. (2019) 46:47–54. doi: 10.1159/000495772 31244581 PMC6558326

[24] Muller T Uherek C Maki G Chow KU Schimpf A Klingemann HG. Expression of a CD20-specific chimeric antigen receptor enhances cytotoxic activity of NK cells and overcomes NK-resistance of lymphoma and leukemia cells. Cancer Immunol Immunother. (2008) 57:411–23. doi: 10.1007/s00262-007-0383-3 PMC11029838 17717662

[25] Lock D Mockel-Tenbrinck N Drechsel K Barth C Mauer D Schaser T. Automated manufacturing of potent CD20-directed chimeric antigen receptor T cells for clinical use. Hum Gene Ther. (2017) 28:914–25. doi: 10.1089/hum.2017.111 28847167

[26] Apel M Brüning M Granzin M Essl M Stuth J Blaschke J. Integrated clinical scale manufacturing system for cellular products derived by magnetic cell separation, centrifugation and cell culture. Chemie Ingenieur Technik. (2013) 85:103–10. doi: 10.1002/cite.201200175

[27] Kaiser AD Assenmacher M Schroder B Meyer M Orentas R Bethke U. Towards a commercial process for the manufacture of genetically modified T cells for therapy. Cancer Gene Ther. (2015) 22:72–8. doi: 10.1038/cgt.2014.78 PMC4356749 25613483

[28] Vucinic V Quaiser A Luckemeier P Fricke S Platzbecker U Koehl U. Production and application of CAR T cells: current and future role of Europe. Front Med. (2021) 8:713401. doi: 10.3389/fmed.2021.713401 PMC8418055 34490302

[29] Palani HK Arunachalam AK Yasar M Venkatraman A Kulkarni U Lionel SA. Decentralized manufacturing of anti CD19 CAR-T cells using CliniMACS Prodigy(R): real-world experience and cost analysis in India. Bone Marrow Transplant. (2023) 58:160–7. doi: 10.1038/s41409-022-01866-5 36347999

[30] Maschan M Caimi PF Reese-Koc J Sanchez GP Sharma AA Molostova O. Multiple site place-of-care manufactured anti-CD19 CAR-T cells induce high remission rates in B-cell Malignancy patients. Nat Commun. (2021) 12:7200. doi: 10.1038/s41467-021-27312-6 34893603 PMC8664838

[31] Schaft N. The landscape of CAR-T cell clinical trials against solid tumors-A comprehensive overview. Cancers. (2020) 12(9):2567. doi: 10.3390/cancers12092567 32916883 PMC7563774

[32] Teoh J Brown LF. Developing lisocabtagene maraleucel chimeric antigen receptor T-cell manufacturing for improved process, product quality and consistency across CD19(+) hematologic indications. Cytotherapy. (2022) 24:962–73. doi: 10.1016/j.jcyt.2022.03.013 35610089

[33] Ghassemi S Durgin JS Nunez-Cruz S Patel J Leferovich J Pinzone M. Rapid manufacturing of non-activated potent CAR T cells. Nat BioMed Eng. (2022) 6:118–28. doi: 10.1038/s41551-021-00842-6 PMC8860360 35190680

[34] Yang J He J Zhang X Li J Wang Z Zhang Y. Next-day manufacture of a novel anti-CD19 CAR-T therapy for B-cell acute lymphoblastic leukemia: first-in-human clinical study. Blood Cancer J. (2022) 12:104. doi: 10.1038/s41408-022-00694-6 35798714 PMC9262977

[35] Zhang C He J Liu L Wang J Wang S Liu L. Novel CD19 chimeric antigen receptor T cells manufactured next-day for acute lymphoblastic leukemia. Blood Cancer J. (2022) 12:96. doi: 10.1038/s41408-022-00688-4 35750687 PMC9232607

[36] Engels B Zhu X Yang J Price A Sohoni A Stein AM. Preservation of T-cell stemness with a novel expansionless CAR-T manufacturing process, which reduces manufacturing time to less than two days, drives enhanced CAR-T cell efficacy. Blood. (2021) 138:2848. doi: 10.1182/blood-2021-146246

[37] Blache U Weiss R Boldt A Kapinsky M Blaudszun AR Quaiser A. Advanced flow cytometry assays for immune monitoring of CAR-T cell applications. Front Immunol. (2021) 12:658314. doi: 10.3389/fimmu.2021.658314 34012442 PMC8127837

[38] Mougiakakos D Kronke G Volkl S Kretschmann S Aigner M Kharboutli S. CD19-targeted CAR T cells in refractory systemic lupus erythematosus. N Engl J Med. (2021) 385:567–9. doi: 10.1056/NEJMc2107725 34347960

[39] Mackensen A Muller F Mougiakakos D Boltz S Wilhelm A Aigner M. Anti-CD19 CAR T cell therapy for refractory systemic lupus erythematosus. Nat Med. (2022) 28:2124–32. doi: 10.1038/s41591-022-02017-5 36109639

[40] Muller F Boeltz S Knitza J Aigner M Volkl S Kharboutli S. CD19-targeted CAR T cells in refractory antisynthetase syndrome. Lancet. (2023) 401:815–8. doi: 10.1016/S0140-6736(23)00023-5 36930673

