# Supplementary figures and images for: Automated manufacturing and characterization of clinical grade autologous CD20 CAR T cells for the treatment of patients with stage III/IV melanoma
Source: Front Immunol. 2024 Sep 25;15:1328368. doi: 10.3389/fimmu.2024.1328368 (PMC11461191; doi:10.3389/fimmu.2024.1328368)

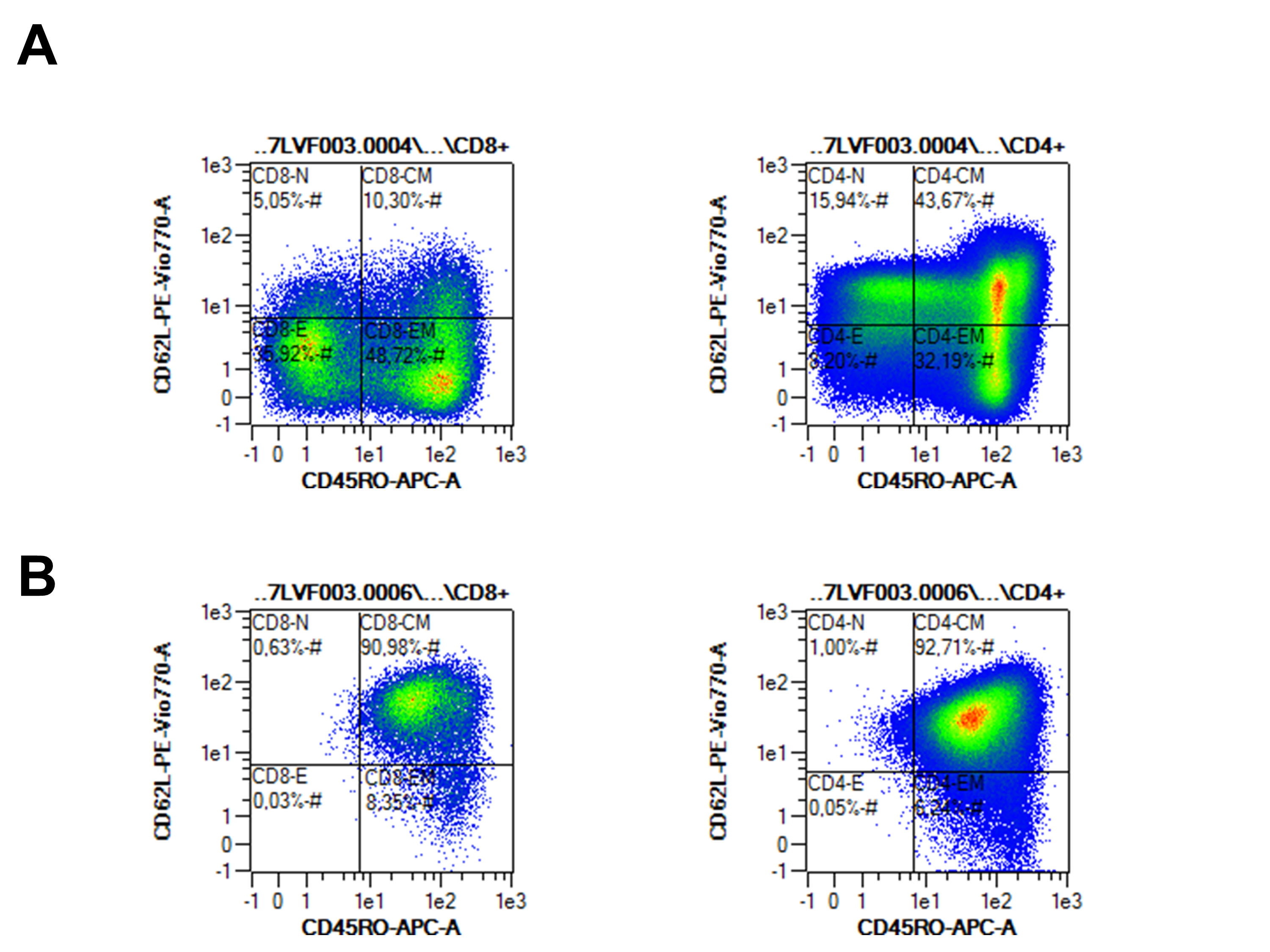

Supplement: Supplementary Figure 1 — Exemplary flow cytometric dot plots on phenotyping of CD8+ and CD4+ cells using CD62L (y-axis) and CD45RO (x-axis) expression. Naïve (N) T cells defined as CD62L+CD45RO-, Central memory (CM) T cells defined as CD62L+CD45RO+, Effector (E) T cells defined as CD62L-CD45RO- and effector memory (EM) defined as CD62L-CD45RO+ are shown on dot plots, gated on CD3+CD8+ (left) or CD3+CD4+ (right) respectively. (A) T cells after CD4/CD8 T cell selection and (B) CAR+ T cells at harvest. [file Image1.tif]
